# DNA Methylation of *COX‐2*, *IFN‐γ*, *TNF‐α*, and LINE‐1 in Clinically Stable Periodontal Tissues Following Periodontal Therapy

**DOI:** 10.1002/cre2.70229

**Published:** 2025-10-07

**Authors:** Giulio Rasperini, Koki Yoshida, Alessandro Martinotti, Valentina Bollati, Letizia Tarantini, Farah Asa'ad

**Affiliations:** ^1^ Department of Biomedical, Surgical and Dental Sciences University of Milan Milan Italy; ^2^ Fondazione IRCCS Ca' Granda Ospedale Maggiore Policlinico Milan Italy; ^3^ Division of Oral Medicine and Pathology, Department of Human Biology and Pathophysiology, School of Dentistry Health Sciences University of Hokkaido Hokkaido Japan; ^4^ School of Medicine, AOU Maggiore della Carità di Novara University of Eastern Piedmont Novara Italy; ^5^ EPIGET‐Epidemiology, Epigenetics and Toxicology Lab, Department of Clinical Sciences and Community Health University of Milan Milan Italy; ^6^ Epidemiology Unit Fondazione IRCCS Ca' Granda Ospedale Maggiore Policlinico Milan Italy; ^7^ Department of Oral Biochemistry, Institute of Odontology The Sahlgrenska Academy at University of Gothenburg Göteborg Sweden

**Keywords:** biomarkers, DNA methylation, epigenetics, inflammation‐related genes, periodontal disease

## Abstract

**Background:**

Epigenetic modifications such as DNA methylation play a crucial role in the regulation of gene expression in inflammatory diseases, including periodontitis. While previous studies have examined methylation changes during active disease or shortly after treatment, little is known about the epigenetic landscape of periodontal tissues that have remained clinically stable over the long term after Supportive Periodontal Therapy (SPT).

**Methods:**

We collected gingival tissue samples from 40 individuals, including 20 with a history of periodontitis currently under long‐term SPT and 20 periodontally healthy controls. DNA methylation levels of LINE‐1 (a marker of global methylation) and inflammation‐related genes *COX‐2* (*PTGS2*), *IFN‐γ* (*IFNG*), and *TNF‐α* (*TNF*) were analyzed using bisulfite pyrosequencing.

**Results:**

The LINE‐1 methylation percentage was significantly higher in the periodontitis group than in the healthy group (66.5% ± 2.0 vs. 63.9% ± 4.0; *p* = 0.03). However, this significance was lost after adjusting for age and gender. No significant differences were observed between groups for *COX‐2*, *IFN‐γ*, or *TNF‐α*. Genomic context analysis using the Encyclopedia of DNA Elements annotations revealed that the CpG sites analyzed for *PTGS2*, *IFNG*, and *TNF* are in distal regulatory regions enriched with enhancer‐like elements, histone modifications, and predicted *NFKB1* binding motifs.

**Conclusions:**

These findings suggest that LINE‐1 methylation in clinically stable gingival tissues may reflect long‐term epigenetic memory from previous chronic inflammation. Motif‐level analysis highlighted potential regulatory input from *NFKB1* at the three loci (*PTGS2*, *IFNG*, and *TNF*). Notably, no significant epigenetic differences were observed in the inflammation‐related genes *COX‐2*, *IFN‐γ*, and *TNF‐α*, suggesting that periodontal disease can be effectively treated and that certain inflammatory markers may return to levels comparable to those seen in individuals who have never had the disease. These results highlight the importance of examining DNA methylation dynamics not only during active disease but also during long‐term remission.

**Abbreviations**
AAP: American academy of periodontology
BED: browser extensible data
BOP: bleeding on probing
bp: base pairs
cCRE: candidate cis‐regulatory element
CpG: cytosine‐phosphate‐guanine
COX‐2: cyclooxygenase‐2
dELS: distal enhancer‐like signature
DNA: deoxyribonucleic acid
ENCODE: Encyclopedia of DNA Elements
H3K27Ac: histone H3 lysine 27 acetylation
IFN‐γ/IFNG: interferon‐gamma
LINE‐1: long interspersed nuclear element‐1
NFKB1: Nuclear Factor Kappa B Subunit 1
NFKB2: Nuclear Factor Kappa B Subunit 2
NSAIDs: non‐steroidal anti‐inflammatory drugs
PD: probing depth
PTGS2: prostaglandin‐endoperoxide synthase 2 (COX‐2 gene symbol)
RSAT: regulatory sequence analysis tools
SPT: supportive periodontal therapy
TNF‐α/TNF: tumor necrosis factor‐alpha
TSS: transcription start site
UCSC: university of california santa cruz (genome browser)

## 1 Introduction

Periodontitis is a chronic inflammatory disease caused mainly by the accumulation of dental plaque, also known as dental biofilm, and is characterized by the destruction of tooth‐supporting structures supporting the teeth (Kwon et al. 2021). The inflammatory host response in periodontitis is influenced by various factors, including genetic predisposition. In addition to variations in genomic sequences that have been associated with chronic inflammatory diseases, there are epigenetic modifications of DNA that are in part acquired during life but can also be inherited and thus can be intrinsic (Loos and Van Dyke 2020). Interestingly, gene expression is modified by epigenetic changes in response to environmental stimuli (Ari et al. 2016). Epigenetics refers to the regulation of gene expression independent of alteration in the DNA sequence, and thus, represents a fundamental link between genetic predispositions and environmental factors. Epigenetics has been shown to contribute to the development and progression of various diseases, including oral inflammatory conditions such as periodontitis and peri‐implantitis (Larsson et al. 2022; Suzuki and Yamada 2022).

Nevertheless, our understanding of oral health from an epigenetics point of view remains limited (Lod et al. 2014; Khouly et al. 2020). Epigenetics induce reversible chemical alterations in both DNA and its associated proteins, called histones, thereby influencing the structure of chromatin and modulating gene activity (Larsson 2017; Asa'ad et al. 2019). Three primary mechanisms govern epigenetic modifications: DNA methylation, histone modifications, and noncoding RNAs (Seo et al. 2015). Therefore, research efforts investigating the connection between epigenetics and periodontal diseases have mainly focused on DNA methylation of genes involved in the regulation of cytokine production, as cytokines play a pivotal role in the breakdown of periodontal tissues (Larsson et al. 2022; Lod et al. 2014; Khouly et al. 2020; Barros and Offenbacher 2014). Additionally, the methylation status of genes associated with cellular signaling pathways has also been explored (Liaw et al. 2022). For instance, de Faria Amormino et al. demonstrated increased methylation in the *TLR2* gene in gingival biopsies from chronic periodontitis patients, linking this methylation to disease severity parameters such as probing depth (PD) and inflammatory cell counts (de Faria Amormino et al. 2013). Similarly, De Oliveira et al. observed alterations in methylation patterns of *TLR2* and *TLR4* genes in periodontitis tissues compared to healthy samples, potentially influencing host–microbe interactions and exacerbating dysbiosis (De Oliveira et al. 2011).

Despite these observations, there is very limited knowledge of the effect of periodontal therapy in restoring normal DNA methylation levels in periodontitis patients. In a previous study by our research group (Asa'ad et al. 2017), we evaluated the effect of nonsurgical periodontal therapy on DNA methylation changes of three genes involved in periodontitis: cyclooxygenase‐2 (*COX‐2* or *PTGS2),* interferon‐gamma (*IFN‐γ* or *IFNG*), and tumor necrosis factor‐alpha (*TNF‐α* or *TNF*), as well as the sequence long interspersed nuclear element‐1 (LINE‐1). In the study, the level of DNA methylation of only *COX‐2* was reset to the levels observed in healthy tissues. However, our study had a short‐term follow‐up: 2 and 8 weeks post‐periodontal therapy.

Periodontitis is a chronic disease that requires ongoing maintenance even after achieving remission through periodontal therapy. In clinical practice, if re‐evaluation after active periodontal treatment reveals that most periodontal tissues are healthy, but a limited number of sites still exhibit PDs of 4 mm or more, slight tooth mobility without signs of inflammation, the condition is clinically stable. Such cases are diagnosed as “stable periodontal condition” and are transitioned into Supportive Periodontal Therapy (SPT). According to Cochrane and American Academy of Periodontology (AAP) guidelines, SPT involves regular periodontal re‐evaluation, risk assessment, and subgingival plaque/calculus removal, with targeted retreatment as needed to maintain long‐term periodontal stability (Manresa et al. 2018).

Although several studies have reported changes in DNA methylation in inflamed periodontal tissues compared to healthy tissues, no research to date has investigated DNA methylation changes in periodontal tissues during the SPT phase (Jurdziński et al. 2020). Exploring the DNA methylation profile of clinically stable periodontal tissues may provide important insights into the mechanisms underlying inflammation recurrence and help in developing preventive strategies.

*COX‐2*, *IFN‐γ*, and *TNF‐α* are inflammation‐related genes that have already been identified as key targets for DNA methylation analysis in periodontitis (Khouly et al. 2020). In addition, LINE‐1, a widely used marker of global DNA methylation, has also been reported to exhibit methylation changes in periodontal disease (Larsson 2017).

While previous studies have investigated DNA methylation changes during the early phases following periodontal treatment, it remains unclear whether such epigenetic modifications persist in clinically stable periodontal tissues after long‐term SPT. Moreover, although *COX‐2*, *IFN‐γ*, *TNF‐α*, and LINE‐1 have been identified as key targets for methylation analysis in the context of active inflammation or disease onset, their methylation status in the long‐term posttreatment phase has not been elucidated. Therefore, the aim of this study was to investigate the DNA methylation patterns of these genes in patients who had transitioned to SPT following a long‐term periodontal treatment to clarify whether epigenetic traces of past periodontitis remain even in clinically stable tissue.

## 2 Methods

### 2.1 Study Participants and Inclusion Criteria

This study was approved by the ethical committee of the University of Milan (ethical approval number: 25/19), Italy, and was conducted between 2020 and 2023.

This study included 40 individuals, divided into two groups of 20 individuals each, resulting in a total of 40 patients. One group consisted of periodontally healthy patients, while the other group comprised patients with periodontitis who had been treated and were currently undergoing SPT.

Patients were selected and enrolled from the pool of patients attending a private clinic in Piacenza, Italy. All patients voluntarily participated in the study after the objectives were explained to them, and verbal and written informed consent was obtained.

### 2.2 Participant Eligibility

All participants enrolled in this study met the following inclusion criteria: at least 18 years of age and self‐identified as being of Caucasian ethnicity. The latter criterion was applied to minimize potential confounding effects related to ethnicity‐associated variations in DNA methylation patterns.

For the healthy group, participants exhibited no clinical signs of gingival inflammation and had no documented history of periodontitis. All periodontal sites demonstrated PDs of ≤ 3 mm, with no evidence of bleeding on probing (BOP), tooth mobility, or other clinical signs of periodontal disease. Most of these individuals were young adults who had undergone third molar (wisdom tooth) extraction or other routine dental procedures.

For the periodontitis group, eligible participants were patients undergoing SPT at the time of sampling, following initial periodontal treatment initiated at least 5 years prior. The diagnosis was based on the 2018 classification system proposed by the AAP and the European Federation of Periodontology (EFP) (Tonetti et al. 2018).

### 2.3 Exclusion Criteria

The exclusion criteria were pregnancy, using non‐steroidal anti‐inflammatory drugs (NSAIDs) within 1 month before study enrollment, and receipt of periodontal therapy within the 3 months preceding enrollment.

### 2.4 Tissue Sampling

As part of the standard periodontal treatment protocol, PD was recorded at six sites around each tooth during the initial periodontal examination. Following nonsurgical periodontal therapy, including scaling and root planning, patients underwent re‐evaluation. Depending on the clinical response, patients either transitioned to SPT for disease prevention or received periodontal surgery, after which they were considered stable and moved into SPT.

Gingival tissue samples for this study were collected during SPT visits that took place more than 5 years after the initial periodontal treatment. After the explanation of the research protocol, the patients had the possibility to address questions and time to think whether to accept participation in the study. Once the informed consent was signed, gingival biopsies, after local anesthesia, were harvested in the maxillary palatal area at the interdental level between the molar and premolar, to reduce discomfort for the patients, using a 3‐mm diameter punch biopsy instrument.

In the healthy control group, biopsies were collected from clinically non‐inflamed sites during the extraction of third molars (wisdom teeth).

In the periodontitis group, gingival biopsies were obtained from clinically stable sites that had previously exhibited signs of inflammation but were considered healthy at the time of sampling. These sites demonstrated a PD of ≤ 4 mm and no BOP. All samples were collected either during clinically indicated periodontal procedures or with informed consent, ensuring minimal additional invasiveness.

### 2.5 DNA Extraction and Bisulfite Conversion

Collected tissue samples were immediately placed into labeled vials containing Allprotect Tissue Reagent (Qiagen, USA) to immediately stabilize nucleic acids, following the manufacturer's recommendations. The samples were then stored at −20°C until DNA analysis. Genomic DNA was isolated using the QIAamp DNA Mini Kit (Qiagen, USA) according to the standard protocol.

For bisulfite conversion, 500 ng of DNA (25 ng/μL) was treated using the EZ DNA Methylation‐Gold Kit (Zymo Research, Orange, California, USA), as per the manufacturer's instructions. The bisulfite‐treated DNA was eluted in 300 μL of M‐Elution Buffer and subsequently used for downstream methylation analysis.

### 2.6 Pyrosequencing

DNA methylation analysis was performed based on previously described protocols (Bollati et al. 2007; Tarantini et al. 2013), with slight modifications. Briefly, a 50 μL PCR mixture was prepared using 25 μL of GoTaq Hot Start Green Master Mix (Promega, Madison, Wisconsin, USA), 1 pmol each of forward and reverse primers, 25 ng of bisulfite‐treated genomic DNA, and nuclease‐free water. The primer sequences used in this study are listed in Table 1.

**Table 1:** Primer sequences and amplification conditions used for DNA methylation analysis.

Target	Forward primer (5′–3)	Reverse primer (5′–3)	Sequencing primer (5′–3)	Analyzed Sequence (5′–3)	Annealing condition	Amplicon size (bp)	Genomic location (Chr/CpG site) |
Global DNA methylation marker							
LINE‐1	TTTTGAGTTAGGTGTGGGATATA	Biotin‐AAAATCAAAAAATTCCCTTTC	AGTTAGGTGTGGGATATAGT	TTC/TGTGGTGC/TGTC/TG	50°C for 30 s	146	
Inflammation‐related gene DNA methylation markers							
*COX‐2*	GGAGATTAGTTTAGAATTGGTTTT	Biotin‐AATCCCCACTCTCCTATCTAATCC	AAGAAGAAAAGATATTTGG	C/TGGAAATTTGTGC/TGTTTGGGGC/TGGTGGAATTC/TGGGG	59°C for 60 s	139	Chr1
							Pos1: 186649540
							Pos2: 186649552
							Pos3: 186649561
							Pos4: 186649570
*IFN‐γ*	Biotin‐GTTTTTTGGATTTGATTAGTTTGA	CAATAACAACCAAAAAAACCCA	TATAACTTATATATTTCATC	G/ATTTCCG/AAAAAAATTAAACC	54°C for 60 s	143	Chr12
							Pos1: 66840192
							Pos2: 66840186
*TNF‐α*	Biotin‐TGAGGGGTATTTTTGATGTTTGT	CCAACAACTACCTTTATATATCCC	ATAAACCCTACACCTTCTAT‐	CTCA/GATTTCTTCTCCATCA/GCA/GAAAACA/GAAAA	57°C for 60 s	208	Chr6
							Pos1:31651172
							Pos2:31651157
							Pos3:31651155
							Pos4: 31651149

*Notes:* This table lists the primers, sequencing information, and amplification conditions used to assess DNA methylation levels at selected CpG sites. LINE‐1 was included as a global DNA methylation marker, while *COX‐2*, *IFN‐γ*, and *TNF‐α* were selected as inflammation‐related genes. Genomic positions for analyzed CpG sites are based on the hg38 human genome reference. Due to the repetitive nature of LINE‐1 elements, specific chromosomal coordinates could not be assigned.

The biotin‐labelled primers were used to purify the final PCR product using Sepharose beads. The PCR product was bound to Streptavidin Sepharose HP beads (Amersham Biosciences, Uppsala, Sweden), and the bead‐bound amplicons were purified, washed, and denatured with 0.2 mol/L NaOH. Further washing was performed using the Pyrosequencing Vacuum Prep Tool (Pyrosequencing Inc., Westborough, Massachusetts, USA) according to the manufacturer's protocol. Then, 0.3 μL of pyrosequencing primer was annealed to the single‐stranded PCR product, and pyrosequencing was then performed using the PyroMark MD system (Pyrosequencing Inc.).

The level of DNA methylation was calculated as the percentage of methylated cytosines relative to the total cytosines at each CpG site (% 5mC). The specific CpG sites analyzed were chosen based on previously published studies evaluating the same genes, as adopted in Asa'ad et al. to ensure methodological consistency and repeatability (Asa'ad et al. 2017; Dawsey et al. 2008; Madrigano et al. 2012; Cantone et al. 2017).

### 2.7 Genomic Position Annotation and Motif Analysis

The chromosomal locations of the analyzed CpG sites for *PTGS2*, *IFNG*, *TNF*, and LINE‐1 were identified using the University of California Santa Cruz (UCSC) Genome Browser (GRCh38/hg38 assembly). For each gene, CpG site coordinates were examined in relation to transcription start sites (TSSs), annotated CpG islands, and Encyclopedia of DNA Elements (ENCODE) cis‐regulatory elements such as distal enhancer‐like signatures (dELSs) (Rosenbloom et al. 2013). Chromatin accessibility and regulatory potential were inferred by referencing histone marks, including Histone H3 lysine 27 acetylation (H3K27Ac) and overlapping annotated regulatory regions.

For transcription factor binding motif analysis, the Regulatory Sequence Analysis Tools (RSAT) matrix‐scan tool (http://embnet.ccg.unam.mx/rsat/matrix-scan_form.cgi) was used. A representative CpG site (designated as “Pos1”) for each gene (*PTGS2*, *IFNG*, and *TNF*) was selected, and a Browser Extensible Data (BED) file covering ±150 bp (total 300 bp) around this site was generated using the UCSC Table Browser and the “Get Sequence” tool. These sequences were analyzed using motif scanning with Nuclear Factor Kappa B (*NFKB*)‐related transcription factor matrices, which have been reported to regulate *PTGS2*, *IFNG*, and *TNF* (Nakayama et al. 2022; Nanbara et al. 2012; Dhungana et al. 2025).

LINE‐1 was excluded from motif analysis because it represents a repetitive transposable element family rather than a single gene with defined regulatory regions (Deininger and Belancio 2016). Its dispersed and highly redundant nature across the genome complicates accurate mapping and interpretation of transcription factor binding motifs in a locus‐specific manner.

The surrounding sequences were used as input in RSAT, and motif scanning was performed using five *NFKB* matrices (MA0105.1–MA0105.4 for *NFKB1* and MA0778.1 for *NFKB2*) from the JASPAR 2024 database (Turatsinze et al. 2008; Medina‐Rivera et al. 2015; Fornes et al. 2020). Scans were conducted using a second‐order Markov background model and a *p* value threshold of 1e–4. The matrix‐scan outputs included matched motifs with strand, position (relative to the CpG site), sequence, and score information.

### 2.8 Statistical Analysis

Analysis of clinical variables between baseline (T0) and follow‐up (T1) in the 20 diseased individuals was performed with the Wilcoxon matched‐pairs signed‐rank test. For the comparison between diseased and healthy individuals, we used the *χ* ^2^ test (for categorical variables) or the Wilcoxon rank‐sum test, also known as the Mann–Whitney test (for quantitative variables). We compared methylation levels between diseased and healthy individuals by fitting multiple linear regression models adjusted for gender and age. Statistical analyses were performed with Stata 19 (StataCorp, USA).

## 3 Results

### 3.1 Sociodemographic Characteristics of the Study Participants

A total of 40 participants (16 males and 24 females) were enrolled in this study and divided into two groups: the diseased group (6 males [30%], 14 females [70%]) and the healthy group (10 males [50%], 10 females [50%]). No statistically significant difference in gender distribution was found between the groups (*p* = 0.20) (Table 2).

**Table 2:** Sociodemographic characteristics of the study participants in the healthy and diseased groups.

Patients' variables	Diseased	Healthy (Control)	*p* value
Mean age (years)	61.4 ± 8.6	49.4 ± 18.0	0.03
Gender	Number (%)		0.20
Male	6 (30%)	10 (50%)	
Female	14 (70%)	10 (50%)	
Smoking habit	Number (%)		0.29
Yes	1 (5%)	3 (15%)	
No	19 (95%)	17 (85%)	
Hygiene sessions' regularity	Number (%)		0.06
Yes	18 (90%)	13 (65%)	
No	2 (10%)	7 (35%)	

*Note:* The table summarizes participants' age, gender distribution, smoking history, and regularity of oral hygiene sessions. A significant age difference was observed between groups (*p* = 0.03), while other variables showed no statistically significant differences.

The mean age in the healthy group was 49.4 ± 18.0 years (range: 21–75 years), while that in the diseased group was significantly higher at 61.4 ± 8.6 years (range: 42–76 years; *p* = 0.03).

Regarding smoking habits, only one participant (5%) in the diseased group and three participants (15%) in the healthy group had a history of smoking. This difference was not statistically significant (*p* = 0.29).

Additionally, regular attendance at hygiene sessions was more frequently observed in the diseased group (90%) than in the healthy group (65%), although the difference did not reach statistical significance (*p* = 0.06).

### 3.2 Clinical Improvements in Periodontitis Patients Over Time

Table 3 summarizes the clinical parameters of periodontitis patients (diseased group) from T0 to the time of biopsy (T1). The mean follow‐up period between T0 and T1 was 10.9 ± 4.0 years.

**Table 3:** Clinical status of 20 patients with periodontitis undergoing long‐term Supportive Periodontal Therapy, at baseline (T0) and follow‐up (T1).

Periodontitis patients' variables (Diseased group)	T0	T1	*p* value
Teeth present			0.00
Total number	516	483	
Mean ± SD	25.8 ± 4.8	24.1 ± 4.6	
Teeth extracted			
Total number	33		
Mean ± SD	1.5 ± 1.7		
Pockets = 5 mm			0.00
Total number	303	38	
Mean ± SD	15.2 ± 8.1	1.9 ± 3.1	
Pockets ≥ 6 mm			0.00
Total number	628	25	
Mean ± SD	31.4 ± 24.1	1.3 ± 2.6	

*Note:* The table summarizes the changes in the number of present teeth, extracted teeth, and periodontal pockets (5 mm and ≥ 6 mm) during the follow‐up period.

At T0, the mean number of present teeth per patient was 25.8 ± 4.8, which decreased to 24.1 ± 4.6 at T1. A total of 33 teeth were extracted over the follow‐up period, corresponding to an average of 1.5 teeth lost per patient.

Although tooth loss was observed, no cases showed progression to a more severe stage according to the 2018 AAP/EFP classification (Tonetti et al. 2018).

Instead, the observed changes in PD and tooth loss between T0 and T1 indicate improvement in periodontal status at the full‐mouth level. In this study, no progression in PD values was observed.

According to the endpoints suggested by Feres et al. and Nibali et al. Stage III or Stage IV periodontitis reflects a history of severe tissue destruction; however, in the absence of clinical inflammation or signs of disease progression under SPT, patients can be considered clinically stable (Feres et al. 2020; Nibali et al. 2020).

The number of sites with a PD of 5 mm was 303 at T0, with a mean of 15.2 ± 8.1 sites per patient (range: 1–39). By T1, this number had decreased to 38 in total, with a mean of 1.9 ± 3.1 sites per patient (range: 0–11), indicating a statistically significant reduction (*p* = 0.00). Similarly, the number of sites with a PD of ≥ 6 mm was 628 at T0, with a mean of 31.4 ± 24.1 sites per patient (range: 3–101). By T1, this number had decreased to 25 in total, with a mean of 1.3 ± 2.6 sites per patient (range: 0–8), indicating a statistically significant reduction (*p* = 0.00).

### 3.3 DNA Methylation Profiles Across Genes

**Figure 1:**
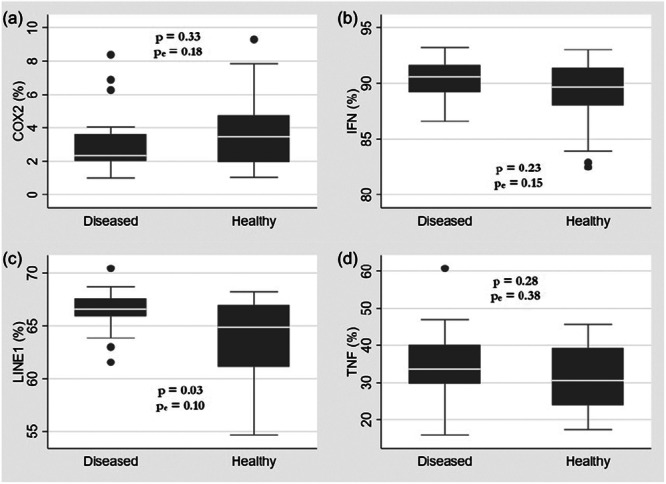
DNA methylation levels of *COX‐2*, *IFN‐γ*, LINE‐1, and *TNF‐α* in diseased and healthy groups. Box plots show the distribution of methylation percentages for each gene. (a, b, d) No significant differences were observed between groups for *COX‐2* (3.1% ± 1.9 vs. 3.8% ± 2.3), *IFN‐γ* (90.4% ± 1.8 vs. 89.1% ± 3.2), or *TNF‐α* (34.8% ± 10.1 vs. 31.3% ± 8.2), even after adjustment for covariates (*p* > 0.05 for all). (c) LINE‐1 exhibited significantly higher methylation in the diseased group (66.5% ± 2.0) compared to the healthy group (63.9% ± 4.0) (*p* = 0.03), though this difference lost statistical significance after adjustment for gender and age (adjusted *p* = 0.10).

DNA methylation levels were compared between the diseased and healthy groups for four targets: LINE‐1, *COX‐2*, *IFN‐γ*, and *TNF‐α* (Figure 1 and Table 4). The LINE‐1 repetitive element showed a significantly higher mean methylation percentage in the diseased group (66.5% ± 2.0) compared to the healthy group (63.9% ± 4.0, *p* = 0.03). However, this difference did not remain statistically significant after adjusting for gender and age covariates (adjusted *p* = 0.10).

**Table 4:** DNA methylation percentages of LINE‐1, *COX‐2*, *IFN‐γ*, and *TNF‐α* genes.

Gene/Sequence	Diseased (%)	Healthy (%)	*p* value	* *p* value adjusted with gender and age as covariates
*COX‐2*			0.33	0.18
Mean ± SD	3.1 ± 1.9	3.8 ± 2.3		
*IFN‐γ*			0.23	0.15
Mean ± SD	90.4 ± 1.8	89.1 ± 3.2		
*TNF‐α*			0.28	0.38
Mean ± SD	34.8 ± 10.1	31.3 ± 8.2		
LINE‐1			0.03	0.10
Mean ± SD	66.5 ± 2.0	63.9 ± 4.0		

*Note:* Mean percentages ± SD values are shown for each group. Statistical comparisons were performed with and without adjustment for gender and age covariates.

* From linear regression models adjusted for gender and age.

For the *COX‐2* gene, both groups exhibited low methylation levels: 3.1% ± 1.9 in the diseased group and 3.8% ± 2.3 in the healthy group (*p* = 0.33, adjusted *p* = 0.18). *IFN‐γ* showed very high methylation levels in both groups (90.4% ± 1.8 vs. 89.1% ± 3.2, *p* = 0.23, adjusted *p* = 0.15), while *TNF‐α* showed moderate methylation levels (34.8% ± 10.1 vs. 31.3% ± 8.2, *p* = 0.28, adjusted *p* = 0.38). None of these gene‐specific differences were statistically significant.

### 3.4 Genomic Context of CpG Sites

**Figure 2:**
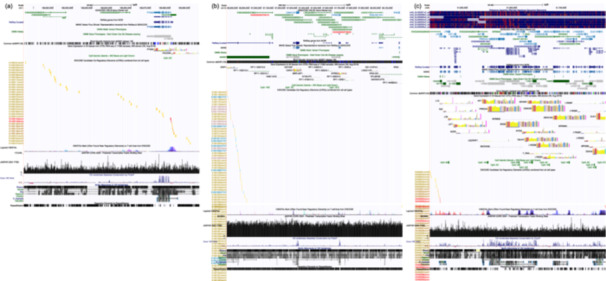
UCSC Genome Browser views of the genomic regions surrounding the analyzed CpG sites in *PTGS2*, *IFNG*, and *TNF*. Tracks include annotated RefSeq genes, CpG islands, candidate cis‐regulatory elements (cCREs), histone modification marks (H3K27Ac), and predicted transcription factor binding sites. A representative figure was not generated for LINE‐1 due to its dispersed and repetitive nature, which prevents accurate genomic localization. However, LINE‐1 methylation was analyzed using consensus primers as a marker of global methylation. (a) The CpG site for *PTGS2* (chr1:186649540) is located approximately 22 kb upstream of the TSS, placing it within a distal enhancer‐like region enriched with H3K27Ac marks and dELS elements. (b) The CpG site for *IFNG* (chr12:66840192) is situated in a gene desert region approximately 1.3 Mb upstream of the *IFNG* TSS and about 1.15 Mb downstream of the TSS of the nearby antisense transcript *IFNG‐AS1*. This intergenic region shows features of potential regulatory activity. (c) The CpG site for *TNF* (chr6:31651172) lies approximately 73 kb downstream of the *TNF* gene body and overlaps with regions annotated as distal regulatory elements marked by H3K27Ac and cCREs.

The CpG site analyzed for *PTGS2* (chr1:186649540) is located approximately 22 kb upstream of the TSS (chr1:186671791), placing it within a non‐CpG island region (Figure 2a). The annotations from the latest UCSC Genome Browser and ENCODE datasets classify it as a distal regulatory region enriched with enhancer‐like features, rather than a canonical proximal promoter. The selected CpG site for *IFNG* (chr12:66840192) lies approximately 1.3 Mb upstream of the *IFNG* TSS (chr12:68159740) and ~1.15 Mb downstream of the *IFNG‐AS1* TSS (chr12:67989447). This region is in a gene desert and does not overlap any annotated CpG islands or promoter regions, suggesting it resides in a distal intergenic context. Nevertheless, its proximity to the *IFNG‐AS1* locus implies possible involvement in long‐range or antisense‐associated regulation (Figure 2b). The *TNF* CpG site (chr6:31651172) is located ~73 kb downstream of the *TNF* transcriptional unit (chr6:31575565–31578336) and resides outside annotated promoter regions (Figure 2c). As LINE‐1 was excluded from the transcription factor motif analysis due to its repetitive nature (Deininger and Belancio 2016), it was also omitted from Figure 2.

### 3.5 Transcription Factor Binding Site Prediction

Using RSAT matrix‐scan and JASPAR 2024 *NFKB* matrices, significant binding motifs were identified within ±150 bp of the representative CpG sites. The results are summarized in Table 5. A total of five significant *NFKB1* binding motifs were identified in *PTGS2*, including three with MA0105.2, and one each with MA0105.1 and MA0105.4. The *IFNG* locus exhibited one significant match for each of the *NFKB1* binding motifs MA0105.1 and MA0105.3. *TNF* exhibited a notably high density of *NFKB1* binding motifs, with four matches for MA0105.1 and seven for MA0105.2, totaling eleven significant hits.

**Table 5:** Motif matches for *NFKB* within ± 150 bp of CpG sites in selected genes.

Target gene	Matrix ID	Transcription factor	Strand	Position (start–end)	Matched sequence	Weight score	*p* value
*PTGS2*	MA0105.2	*NFKB1*	R	−47 to −37	GGGGTTTCGCC	9.7	1.00E−05
	MA0105.1	*NFKB1*	R	−46 to −37	GGGGTTTCGC	9.7	1.10E−05
	MA0105.2	*NFKB1*	R	−93 to −83	GGTGATCCGCC	7.6	6.10E−05
	MA0105.4	*NFKB1*	R	−94 to −82	AGGTGATCCGCCC	3.1	6.40E−05
	MA0105.2	*NFKB1*	R	−46 to −36	TGGGGTTTCGC	7.2	8.30E−05
*IFNG*	MA0105.1	*NFKB1*	R	−194 to −185	GGGACATTTT	7.1	8.00E−05
	MA0105.3	*NFKB1*	R	−168 to −158	GAGATTTTCCT	7	9.60E−05
*TNF*	MA0105.1	*NFKB1*	R	−147 to −138	GGGAGCGTCT	11.4	1.50E−06
	MA0105.2	*NFKB1*	R	−149 to −139	GGAGCGTCTGC	9.5	8.80E−06
	MA0105.2	*NFKB1*	R	−148 to −138	GGGAGCGTCTG	8.6	2.50E−05
	MA0105.1	*NFKB1*	R	−94 to −85	GGGTCTGTAG	8.5	3.40E−05
	MA0105.2	*NFKB1*	R	−93 to −83	GGGGGTCTGTA	8.3	3.70E−05
	MA0105.1	*NFKB1*	R	−148 to −139	GGAGCGTCTG	7.6	5.10E−05
	MA0105.2	*NFKB1*	R	−146 to −136	GAGGGAGCGTC	7.6	6.00E−05
	MA0105.2	*NFKB1*	R	−147 to −137	AGGGAGCGTCT	7.4	6.60E−05
	MA0105.1	*NFKB1*	R	−67 to −58	GTGGCGTCTG	7.3	7.10E−05
	MA0105.2	*NFKB1*	R	−68 to −58	GTGGCGTCTGA	7.2	8.00E−05
	MA0105.2	*NFKB1*	R	−150 to −140	GAGCGTCTGCT	6.9	1.00E−04

*Note: NFKB* binding motifs were identified using RSAT matrix‐scan with JASPAR 2024 matrices (MA0105.1–MA0105.4 and MA0778.1). Positions are relative to the CpG site (0 = CpG position). Only matches with *p* < 1e–4 are shown.

## 4 Discussion

In this study, we compared the DNA methylation patterns of inflammation‐related genes (*COX‐2*, *IFN‐γ*, and *TNF‐α*) and LINE‐1, a marker of global DNA methylation, in gingival tissues from patients who had undergone over 5 years of periodontal treatment and were in a clinically stable condition under SPT, and from healthy individuals. LINE‐1 methylation was initially higher in the diseased group, but this difference was no longer statistically significant after adjusting for age and gender covariates.

Global DNA methylation, including LINE‐1, is generally known to decline with aging, reflecting genomic instability and reduced maintenance of epigenetic regulation (Erichsen et al. 2018). However, a previous study has shown that patients with a history of aggressive periodontitis exhibited higher LINE‐1 methylation in oral epithelial cells than healthy controls (Baptista et al. 2014). This suggests that strong inflammation may elevate LINE‐1 methylation, possibly as a protective or adaptive epigenetic response.

In the present study, LINE‐1 methylation was higher in the older periodontitis group than in the younger healthy controls, which runs counter to the typical age‐associated trend. This finding may reflect an epigenetic memory of past inflammation that partially counteracts the age‐related decline in methylation. However, although the difference was not statistically significant after covariate adjustment, the possibility that age‐related factors underlie this pattern cannot be ruled out. Therefore, both inflammation and aging may contribute to LINE‐1 methylation levels, and the effect of either factor alone cannot be conclusively determined in this study.

Our previous study, which focused on the short‐term post‐treatment phase (2–8 weeks), found no significant changes in LINE‐1 methylation (Asa'ad et al. 2017). In contrast, the current findings suggest that LINE‐1 may serve as an epigenetic record of chronic periodontal inflammation that persists even after clinical resolution.

Nevertheless, a major limitation of the present study is that the age distributions of the diseased and control groups were not matched. Furthermore, LINE‐1 methylation could not be compared between the acute and remission phases of inflammation. Because reports of inflammation‐induced changes in LINE‐1 methylation remain scarce even outside periodontology, these findings should be interpreted with caution, and further investigation is warranted.

In this study, we analyzed the DNA methylation status of *COX‐2*, *IFN‐γ*, and *TNF‐α*, which are genes already identified as relevant targets for methylation evaluation in periodontitis (Khouly et al. 2020). In periodontal tissues that had clinically stabilized following long‐term periodontal treatment and transitioned to SPT, no significant differences in methylation levels of these genes were found between the healthy and diseased groups.

Our previous study showed that *COX‐2* methylation levels were significantly higher in the periodontitis group, before short‐term periodontal therapy (Asa'ad et al. 2017). This finding is consistent with the report by Zhang et al. which demonstrated that *COX‐2* exhibits hypermethylation and reduced mRNA expression under chronic inflammatory conditions (Zhang et al. 2010a). Since DNA methylation is a reversible epigenetic modification (Lu et al. 2006), the present findings suggest that the methylation status of *COX‐2* may return to a healthy level once inflammation is resolved, providing evidence of epigenetic plasticity during periodontal tissue healing.

Two previous studies regarding *IFN‐γ* have reported no significant differences in methylation levels between periodontitis and healthy tissues (Asa'ad et al. 2017; Viana et al. 2011), while one study reported decreased methylation in periodontitis tissues (Zhang et al. 2010b). For *TNF‐α*, findings from three studies are inconsistent: one showed significantly increased methylation, one showed decreased methylation, and the other found no difference between groups (Asa'ad et al. 2017; Zhang et al. 2013; Lavu et al. 2019). Given the limited number of studies, drawing definitive conclusions remains difficult. However, the lack of significant differences in *IFN‐γ* and *TNF‐α* methylation in our current analysis suggests that in clinically stable periodontal tissues, where active inflammation is absent, there may be no pronounced methylation changes in these genes. Further studies are warranted to confirm these observations.

Our analysis revealed that the CpG sites investigated are located outside canonical promoter or CpG island regions, yet reside in chromatin contexts enriched with regulatory features. The CpG site in *PTGS2* is located within a distal regulatory region characterized by enhancer‐like features, including multiple dELS and H3K27Ac marks. It lies approximately 22 kb upstream of the TSS, placing it well outside the canonical promoter region. Several significant matches for *NFKB1* binding motifs were also detected at this site, suggesting a potential role in long‐range transcriptional regulation. The *IFNG* CpG site, located approximately 1.3 Mb upstream of the IFNG TSS and over 1 Mb downstream of *IFNG‐AS1*, resides in a gene desert and showed significant matches for *NFKB1* binding motifs. This suggests potential involvement in antisense‐mediated regulation or distal enhancer activity. The *TNF* CpG site, situated ~73 kb downstream of the gene body, exhibited the highest number of *NFKB1* motif matches among the three loci and coincided with enhancer‐like chromatin features. These findings imply a potential regulatory function that operates independently of promoter‐localized methylation.

The positional classification of CpG sites in the genome is typically defined relative to CpG islands, starting with the CpG island itself, followed by north and south shores ( ~2 kb flanking regions), north and south shelves (~2–4 kb outside the shores), and OpenSea regions, which lie > 6 kb away from any CpG island (Jones 2012; Shen et al. 2013). All the CpG sites analyzed in the present study, including those in *PTGS2*, *IFNG*, and *TNF*, are located in OpenSea regions far from gene bodies or proximal promoters. These regions, although traditionally considered nonfunctional, are increasingly recognized for their roles in gene regulation, especially in control regions such as enhancers and insulators located outside of TSSs (Jones 2012). However, DNA methylation studies focusing specifically on OpenSea regions remain limited, and further research is needed to elucidate their biological significance. These results emphasize that CpG methylation occurring far from gene bodies or promoters, particularly within OpenSea regions, can still play biologically meaningful regulatory roles. This is especially relevant in chronic inflammatory conditions such as periodontitis, where transcriptional regulation involving key factors like *NFKB* may be mediated via distal regulatory elements. To our knowledge, while several studies have investigated methylation patterns at inflamed sites or shortly after clinical resolution (Khouly et al. 2020; Asa'ad et al. 2017; Andia et al. 2015), this is the first study to focus on long‐term methylation changes following resolution of chronic periodontal inflammation.

These findings emphasize the importance of studying DNA methylation dynamics not only during active disease and immediate post‐treatment phases but also in long‐term maintenance periods, which have been understudied. Such studies would provide deeper insights into the epigenetic mechanisms underlying periodontal disease and its long‐term remission phase.

This study had several limitations. Due to the limited availability of patient‐derived gingival tissue samples, it was not possible to assess correlations between DNA methylation changes and corresponding mRNA expression levels. For the same reason, the analysis was restricted to a limited number of target genes, and comprehensive genome‐wide profiling of DNA methylation could not be performed. Moreover, the current analysis did not evaluate correlations between DNA methylation levels and clinical periodontal parameters, which limits our ability to interpret the functional significance of the observed methylation patterns.

Furthermore, a priori power analysis based on our previous study suggested that a substantially larger sample size, specifically, more than 100 subjects per group, would be required to reliably detect the observed differences (Asa'ad et al. 2017). However, due to clinical and logistical constraints, collecting such a large number of gingival tissue samples was not feasible. This limitation should be considered when interpreting the results.

Another limitation is that the analyzed samples included a mixture of gingival epithelium and underlying connective tissue, making it difficult to determine the specific tissue origin of the methylation changes.

Future investigations should employ genome‐wide approaches such as omics‐based analyses to identify disease‐specific DNA methylation signatures in periodontal tissues maintained in a clinically stable condition after inflammation has resolved (Kebschull et al. 2023). In addition, advanced techniques such as laser microdissection or single‐cell analysis may allow for more precise, tissue‐ or cell‐type‐specific epigenetic profiling.

## 5 Conclusions

This study demonstrated that LINE‐1, a marker of global DNA methylation, exhibited elevated methylation levels in clinically stable gingival tissues from patients with a history of periodontitis. Although *COX‐2* (*PTGS2*), *IFN‐γ* (*IFNG*), and *TNF‐α* (*TNF*) did not show significant methylation differences between groups, genomic analyses revealed that the CpG sites examined reside in regulatory regions with transcriptional potential. These areas contained enhancer‐like features and multiple matches to *NFKB1* binding motifs, suggesting they may retain regulatory capacity even in the absence of methylation differences. These findings highlight the potential of DNA methylation signatures to capture lasting epigenetic imprints of previous inflammatory activity and emphasize the need for comprehensive genome‐wide studies to uncover persistent epigenetic alterations in clinically stable tissues after periodontitis. Importantly, the absence of significant differences in inflammatory gene methylation between the healthy group and the periodontitis group undergoing SPT suggests that periodontal therapy may help restore the epigenetic landscape to a condition similar to that of periodontally healthy individuals. This emphasizes the potential clinical impact of periodontal therapy in not only resolving inflammation but also modulating epigenetic states toward a healthy baseline.

## Author Contributions

G.R. conceived and designed the study. G.R. collected the clinical samples. V.B. and L.T. performed DNA extraction, bisulfite treatment, and the methylation analysis. K.Y. performed the bioinformatics analyses. A.M., K.Y. and F.A. drafted the manuscript. K.Y. and F.A. interpreted the data and critically revised the manuscript. All authors read and approved the final manuscript.

## Ethics Statement

This study was approved by the ethical committee of the University of Milan (ethical approval number: 25/19).

## Consent

All study participants have provided verbal and written consent.

## Conflicts of Interest

The authors declare no conflicts of interest.

## Data Availability

The datasets generated and/or analysed during the current study are not publicly available due to restrictions related to participant privacy and ethical approval. However, the data may be available from the corresponding author upon reasonable request.
